# CDC’s Emergency Management Program Activities — Worldwide, 2013–2018

**DOI:** 10.15585/mmwr.mm7002a2

**Published:** 2021-01-15

**Authors:** Adriana Rico, Cecelia A. Sanders, Amber S. Broughton, Melanie Andrews, Francis A. Bader, David L. Maples

**Affiliations:** ^1^Division of Emergency Operations, Center for Preparedness and Response, CDC; ^2^Office of the Director, Center for Preparedness and Response, CDC; ^3^Oak Ridge Institute for Science and Education, Oak Ridge, Tennessee.

CDC continually evaluates its Emergency Management Program (EMP) activities, including Incident Management System (IMS) activations, use of EMP functions (referred to as EMP utilizations), and exercises, to ensure that the agency is ready to respond to infectious disease outbreaks, disasters (human-made or natural), and security events. Such evaluation not only documents baseline preparedness and response activities during a selected analytical period, but also highlights significant EMP actions that can guide and inform future emergency operations. To characterize EMP activities that occurred during January 1, 2013–December 31, 2018, CDC conducted a retrospective analysis of operational activity logs. The results showed 253 domestic (U.S. states and territories) and international EMP activities, including 12 IMS activations, 147 EMP utilizations, and 94 exercises. Infectious diseases were the most common threat among both IMS activations (58%) and EMP utilizations (52%). CDC responded to the 2014 Ebola epidemic and the 2016 Zika outbreak; each response lasted approximately 2 years and required extended collaboration with domestic and international partners. Understanding the trends in EMP activities, including knowing the most common threats, aids CDC in allocating resources and focusing preparedness efforts. In 2013, CDC became the first federal agency to receive full agency-wide accreditation by the Emergency Management Accreditation Program (EMAP) in recognition of CDC’s commitment to preparedness and its ability to respond to domestic and global public health threats. CDC received EMAP reaccreditation in December 2018 (*1*,*2*).

CDC first implemented the IMS in 2005 based on lessons learned from the Hurricane Katrina response and has since used the IMS as the standard for responding to public health threats (*3*,*4*). CDC activates an agency-level IMS when CDC leadership approves a recommendation from a Preliminary Assessment Team (PAT) comprising program and emergency response subject matter experts. PAT assesses the situation, determines that a program has exhausted available resources, and recommends an agency-level activation to provide enhanced coordination of operations and resources (e.g., staff members, deployment support, equipment, and systems) across the agency. During the period covered in this analysis, CDC IMS activations ranged from level 1, the highest level of activation, to level 3, the lowest level. In other instances, when an IMS activation was not needed but specific support was required, CDC used EMP utilizations by providing technical assistance, including use of the Emergency Operations Center, developing plans and situational reports, distributing emergency public health messages, assisting with data analysis, or providing deployment travel assistance for CDC staff members.

During January 1, 2013–December 31, 2018, CDC conducted a variety of exercises as part of preparedness efforts to ensure that plans and processes were operationally defined should a public health event occur. Exercise types included drills (testing a single response function), full-scale (deployment of resources mimicking a real emergency), functional (exercising a specific IMS element), and tabletop exercises (discussion of a scenario). To further characterize IMS activations, EMP utilizations, and exercises, CDC defined and categorized each event as one of the following: an adverse event (an event resulting in unexpected harm, injury, or illness caused by exposure to a medication, vaccine, or medical equipment or procedure); an infectious disease (an event involving a disease caused by the introduction of a pathogenic agent or microorganism into the body); a human-made event (an event caused directly or principally by human intent, error, or neglect); a mass gathering (an event with large social crowds); a natural disaster (an event related to an environmental cause such as weather or physical characteristics of an area); a national security event (a large gathering involving political and government leaders, delegates, or emissaries), a nuclear/radiological event (involving exposure to nuclear or radiological agents); substance abuse (involving the misuse of prescription drugs or illicit drugs); or other event (not related to defined categories).

During 2013–2018, CDC conducted 253 domestic and international EMP activities, including 12 IMS activations, 147 EMP utilizations, and 94 exercises (Table). IMS activations (58%) and EMP utilizations (52%) were prompted most frequently by infectious disease, followed by human-made events and natural disasters (both 17%) for IMS activations, and human-made events for EMP utilizations (29%). The majority of EMP activities occurred domestically (221, 87%), and EMP utilizations occurred most frequently (147, 58%). Among EMP utilizations, two involved substance-abuse threats; both included distribution of a Health Alert Network notice, a vital public health incident message. Among exercises, six (6%) were large functional exercises conducted to test CDC preparedness for threats such as infectious disease outbreaks including a pandemic influenza, human-made event, natural disaster, and a nuclear/radiological event.

**TABLE:** Number of Emergency Management Program activities (N = 253), by type, cause, and location — Emergency Management Program, CDC, 2013–2018

Activity type/cause (%)	Location			Total
	Domestic	International	Both	
**IMS activations**				
Adverse event (none)	—	—	—	**—**
Infectious disease (58)	1	3	3	**7**
Human-made event (17)	1	—	1	**2**
Mass gathering (none)	—	—	—	**—**
Natural disaster (17)	2	—	—	**2**
National security event (none)	—	—	—	**—**
Nuclear/Radiological (none)	—	—	—	**—**
Other (8)	1	—	—	**1**
Substance abuse (none)	—	—	—	**—**
**Total**	**5**	**3**	**4**	**12**
**EMP utilizations**				
Adverse event (7)	9	1	—	**10**
Infectious disease (52)	72	4	—	**76**
Human-made event (29)	25	17	—	**42**
Mass gathering (1)	2	—	—	**2**
Natural disaster (3)	2	2	—	**4**
National security event (5)	7	—	—	**7**
Nuclear/Radiological (<1)	1	—	—	**1**
Other (2%)	3	—	—	**3**
Substance abuse (1)	2	—	—	**2**
**Total**	**123**	**24**	—	**147**
**Exercises**				
**Drills (82)**	**77**	**—**	**—**	**77**
**Full-scale (10)**				
Infectious disease	1	1	—	**2**
Human-made event	1	—	—	**1**
Natural disaster	4	—	—	**4**
Nuclear/Radiological	2	—	—	**2**
**Functional (6)**				
Infectious disease	3	—	—	**3**
Human-made event	1	—	—	**1**
Natural disaster	1	—	—	**1**
Nuclear/Radiological	1	—	—	**1**
**Tabletop (2)**				
Natural disaster	1	—	—	**1**
Nuclear/Radiological	1	—	—	**1**
**Total**	**93**	**1**	**—**	**94**

**Abbreviations:** EMP = Emergency Management Program, IMS = Incident Management System.

## Incident Management System (IMS) Activations

Although nine of the 12 IMS activations during 2013–2018 were wholly or partially domestic responses, an increase occurred in international responses and in those having both a domestic and international impact. The proportion of activations that included international involvement increased from 14 of 55 during 2003–2012 to seven of 12 during 2013–2018 (*3*). Nine IMS activations during 2013–2018 were conducted at a level 3, one at level 2, and two at level 1 (Figure). The level 1 events (the 2014 Ebola Response and the 2016 Zika Response) had an impact on public health systems domestically and internationally. As the responses intensified, the activation levels also increased; activation levels declined with response de-escalation. Apart from the Polio Response, which has been ongoing since 2011, the longest IMS activations during this timeframe were the 2014 Ebola Response (level 1, 632 days; 3,285 total domestic and international field deployments of CDC staff members) followed by the 2016 Zika Response (level 1, 617 days; 1,718 total domestic and international field deployments of CDC staff members). The shortest IMS activation was the 2018 Hurricane Florence Response, lasting 17 days at level 3. CDC also faced a new type of public health response in 2014, when a substantially higher-than-usual number of unaccompanied immigrant children crossed the southern border into the United States, prompting a level 3 IMS activation. During 2016, CDC responded to four events simultaneously through IMS activations: 2011 Polio, 2014 Ebola, 2016 Zika, and 2016 Flint Water Contamination Response (Figure).

**FIGURE:**
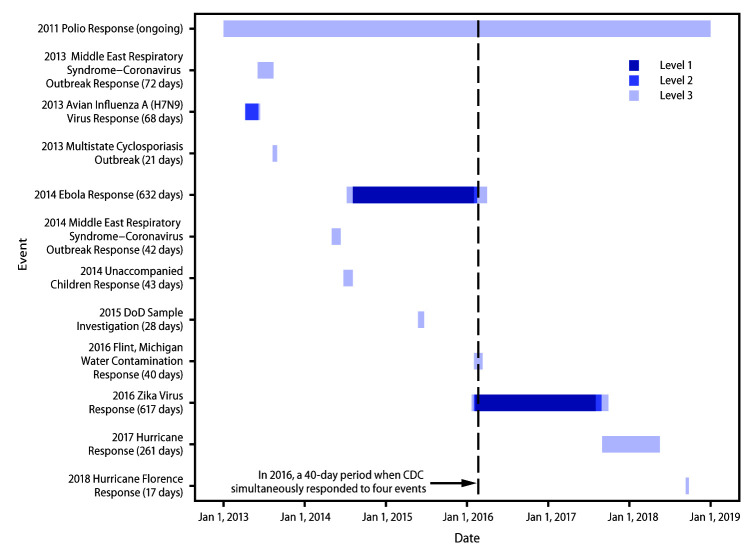
Incident Management System (IMS) activations (N = 12),* by date, duration (in number of days), and activation level^†^ — Emergency Management Program, CDC, 2013–2018 **Abbreviations: **DoD = Department of Defense; Ebola = Ebola virus disease; polio = poliovirus. * Total duration of IMS activation (in days) denoted in parentheses. Year in response name indicates the year that the event was initiated. ^^† ^^ Level 1 is the highest level of activation, requiring a 24/7 agency-wide effort. Level 2 involves a large number of staff members from the relevant program areas and from the Emergency Operations Center (EOC), and time-sensitive tasks and needs might extend beyond core business hours. Level 3 is the lowest level of activation, in which CDC subject matter experts lead the response with their program staff members and assistance from the EOC.

## Discussion

This analysis demonstrated an overall increase in CDC EMP activities (IMS activations, EMP utilizations, and exercises), from 194 during the 10-year period 2003–2012 to 253 during the 6-year period 2013–2018 (*3*). CDC’s EMP has responded to more international activities in the last 6 years than previously reported (*3*). International events can be resource-intensive and require more extensive and expanded coordination within CDC (among CDC and field staff members), with other government entities, and with external partners, adding multiple layers of complexity.

This analysis highlighted two back-to-back international responses (2014 Ebola and 2016 Zika) lasting approximately 4 of the 6 years assessed. These events required simultaneous response activities in multiple countries and rostering of staff members with a range of technical skills including the ability to speak languages other than English (*5*). Deploying staff members with technical expertise combined with foreign-language skills is critical in meeting response demands. International IMS activations present additional challenges, including limited infrastructures, weak health care systems, security threats, political instabilities, and cultural challenges in the affected countries. For example, security challenges in countries with endemic polio transmission have complicated deployment of CDC staff members (*6*).

Today, CDC continues simultaneous responses to several ongoing domestic and international public health emergencies and must be prepared to counter other novel or unconventional public health threats as they occur. Examination of how the agency has addressed such emerging hazards over the analysis period highlights both the increasing complexity of responses and several opportunities for applying lessons learned to current and future response operations. Although the 2016 Zika Response did not involve a novel virus, congenital microcephaly and newborn brain abnormalities associated with Zika virus infection during pregnancy were new, and the route of sexual transmission was previously unknown (*7*). CDC responded quickly to provide guidance to health care professionals and the public on the prevention of Zika virus infection and to issue guidance for laboratory testing. The 2014 Unaccompanied Children Response demonstrated that unconventional situations require CDC to adapt quickly and support other U.S. government entities (e.g., Department of Homeland Security, Administration for Children and Families, and Assistant Secretary for Preparedness and Response) by providing public health technical assistance.

Although CDC responds to various public health events, preparedness efforts are equally important; conducting regular exercises and increasing preparedness planning are critical to mitigating risks and responding to threats. CDC conducts agency-wide exercises and participates in exercises led by other U.S. agencies to enhance CDC’s role in providing public health expertise. As part of CDC’s preparedness efforts, CDC received full agency-wide accreditation by EMAP in 2013, becoming the first federal public health agency to achieve this status and then received reaccreditation in 2018. This accreditation process not only serves as an external evaluation but also requires CDC to review its preparedness for responding to a prioritized list of public health threats in a structured way to implement standard processes and procedures. Understanding common threats and what is required to respond in addition to having standard processes and procedures has improved CDC’s preparedness for responding to these threats. Furthermore, lessons learned from these events have enabled CDC to apply and adapt the IMS to unconventional or novel threats.

**Summary**

**What is already known about this topic?**

CDC’s Emergency Management Program (EMP) uses the Incident Management System (IMS) to respond to public health emergencies and provides technical assistance by applying emergency management principles to public health responses and exercises.

**What is added by this report?**

During 2013–2018, CDC conducted 12 IMS activations, 147 EMP utilizations, and 94 exercises, an increase from the previous 10 years. In 2018, CDC was reaccredited by the Emergency Management Accreditation Program, highlighting CDC’s preparedness to respond to various hazards and global public health threats.

**What are the implications for public health practice?**

As more complex and novel public health emergencies occur, CDC, other agencies, and programs can use and adapt the IMS to respond to these events.
